# Importance of Redox Equilibrium in the Pathogenesis of Psoriasis—Impact of Antioxidant-Rich Diet

**DOI:** 10.3390/nu12061841

**Published:** 2020-06-20

**Authors:** Anna Winiarska-Mieczan, Tomasz Mieczan, Grzegorz Wójcik

**Affiliations:** 1 Department of Bromatology and Food Physiology, University of Life Sciences in Lublin, 20-950 Lublin, Poland; anna.mieczan@up.lublin.pl; 2 Department of Hydrobiology and Protection of Ecosystems, University of Life Sciences in Lublin, 20-262 Lublin, Poland; 3 Department of Inorganic Chemistry, Maria Curie-Sklodowska University, 20-031 Lublin, Poland; g.wojcik@poczta.umcs.lublin.pl

**Keywords:** psoriasis, exogenous antioxidants, diet, prevention, perspectives

## Abstract

Psoriasis is a common, chronic, hyperproliferative, inflammatory skin disease occurring in most ethnic groups in the world. The disease is hereditary but the process of its inheritance is complex and still not fully understood. At the same time, it has been observed that psoriatic lesions may be triggered by certain prooxidative external factors: using narcotics, smoking, drinking alcohol, physical and mental stress, as well as bacterial infections and injury. Since the main physiological marker of psoriasis relates to disorders in the organism’s antioxidative system, it is necessary to develop a well-balanced combination of pharmaceuticals and dietary antioxidants to facilitate the effective treatment and/or prevention of the disease. The dietary sources of antioxidants must be adequate for chronic use regardless of the patient’s age and be easily available, e.g., as ingredients of regular food or dietary supplements. Diet manipulation is a promising therapeutic approach in the context of modulating the incidence of chronic diseases. Another potentially viable method entails the use of nutrigenomics, which guarantees a multiaspectual approach to the problem, including, in particular, analyses of the genetic profiles of psoriasis patients with the view to more accurately targeting key problems. The present paper pertains to the significance of redox equilibrium in the context of psoriasis. Based on information published in worldwide literature over the last decade, the impact of dietary exogenous antioxidants on the course of this chronic disease was analysed.

## 1. Introduction

Psoriasis is a common, chronic, hyperproliferative, inflammatory skin disease occurring in most ethnic groups worldwide, occurring in approximately 2–3% of the population [1,2]. Psoriasis affects both male and female patients, in approximately 75% of the cases developing before the age of 40. The primary underlying cause of psoriasis stems from genetic predispositions [1,3]. Nonetheless, other factors have also been observed to impact the development of the disease, including dermal and systemic overexpression of proinflammatory cytokines, particularly interleukins (IL), tumour necrosis factor (TNF), and interferon-γ (IFN-γ), as well as complex molecular interactions between epidermal keratinocytes, mononuclear leukocytes, neutrophils, dendritic cells, and activated T-cells [2].

Psoriasis is a hereditary condition but the process of its inheritance is complex and still not fully understood. Over 60 genes have been identified as factors conditioning the disease, with a particular focus on the pathogenic contribution of genes related to the activation of Th17 cells [4]. A number of genes take part in the inheritance process and yet, even in the presence of the particular gene combination, psoriasis may not develop. At the same time, it has been observed that psoriatic lesions can be triggered by certain external factors—e.g., using narcotics, smoking, drinking alcohol, physical and mental stress, as well as bacterial infections or injury—due to the resulting gene–environment reactions [2,3]. The mentioned factors are common causes of strong oxidative stress [3,5,6,7], which leads to the conclusion that a disturbance of the organism’s oxidative balance constitutes one of the primary causes of psoriasis in people with certain genetic predispositions. Moreover, activation of nicotinic pathways can increase the production of cytokines such as IL-12, IL-2, TNF, INF-α, and factors stimulating the formation of granulocyte colonies [8].

Oxidative stress can be a direct or indirect risk factor related to various disorders and diseases. It is also known to exacerbate already existing conditions. There is evidence to suggest that excessive amounts of reactive oxygen species (ROS) intermediating the emergence of oxidative stress, take part in numerous biological reactions that trigger DNA modifications and enhance lipid peroxidation and production of inflammatory cytokines [2,9]. Due to the upset process of terminal keratinocyte differentiation, psoriatic epidermis is unable to properly stratify; usually, the granular layer is absent while the stratum corneum contains nucleated cells [10]. T-cells and dendric cells infiltrate into the resulting lesions and produce proinflammatory cytokines. The interaction between activated T-lymphocytes and keratinocytes triggers chronic inflammation. The oxidative stress emerging in undifferentiated keratinocytes leads to the formation of abnormal stratum corneum as a result of inflammation, which increases the antioxidative activity and overexpression of apoptotic receptors [11]. In the course of psoriasis, one observes elevated levels of myeloid dendric cells in the skin, likely forming in response to the release of alpha interferon (IFN-α) as well as other proinflammatory cytokines and chemokines [12,13]. Myeloid dendric cells produce a number of proinflammatory cytokines such as TNF or IL [10]. As the outermost protective layer of the organism, the skin is more susceptible to excessive UV exposure compared to other tissues. UV radiation additionally contributes to decomposition of oxygen molecules into free atoms which can subsequently react with other oxygen molecules producing ozone—a reactive oxygen species [14]. Due to excitation, highly reactive singlet oxygen is formed. ROS created through the skin’s exposure to UV radiation can trigger inflammation leading to cell damage. It is estimated that the effects of UV radiation on skin are a major source of ROS, second only to respiratory chains [15]. This is one of the reasons why skin is particularly susceptible to the harmful effects of ROS [2]. At the same time, a therapy using controlled doses of UV can be used in psoriasis treatment [16]. It has been demonstrated that damage to the basal layer of epidermis affected by psoriatic lesions is likely related to defects of the α-1-antitrypsin inhibitor and the disturbed balance between tissue proteolytic enzymes and their inhibitors [17]. Increased levels of α-2-macroglobulin due to lowered oxygen metabolism have also been observed in the course of psoriasis [18]. The phenomenon can be explained by reduced inactivation of protease inhibitors in the blood serum caused by the increased generation of ROS. Moreover, pruritus experienced in the locations of psoriatic lesions suggests the emergence of neurogenic inflammation related to ROS, similarly to neurological inflammatory disorders [19].

Free radicals originate primarily from mitochondria where, in the process of aerobic metabolism, oxygen is reduced to water [20]. One of the primary means of metabolite exchange between mitochondria and cytoplasm is the VDAC (voltage-dependent anion selective channel) intermediating in the determination of the oxidoreductive status of cytosol [21]. Cytosol is an important factor controlling the synthesis level of, for example, proteins eliminating the superoxide anion radical [21]. Damage induced by reactive oxygen and nitrogen species within mitochondrial DNA (mtDNA) is the source of mitochondrial mutagenesis [22]. The high susceptibility of mtDNA to oxidative damage is due to the fact that it is found in close proximity to the main source of ROS, i.e., the respiratory chain, as well as to the lack of histone protection in mtDNA. This may stem from the absence of intones, which, given the simultaneously high density of information contained in mtDNA, means that damage can occur at any given point of the mitochondrial genome. At the same time, limited capacity was observed for repairing mtDNA and proteins damaged due to replication errors related to oxidative phosphorylation [20]. Studies suggest that some nutrients may offer protection against oxidative damage to mitochondria, including in particular ω3 fatty acids, vitamin C, vitamins from the B group, folic acid, as well as antioxidative minerals such as zinc or magnesium [23].

Psoriasis is an incurable disease; treatment can only reduce the severity of its symptoms. Although it has been fairly well characterised clinically and histologically, its pathogenesis has yet to be fully explained, which is necessary if effective treatment and prophylactics are to be developed. As the main physiological marker of psoriasis is related to functional disorders of the antioxidative system [2,24,25,26], the primary method of supporting pharmacological treatment ought to entail the use of exogenous antioxidants, particularly those suitable for long-term use irrespective of the patient’s age, and be readily available, for example, being present in food or diet supplements. The paper analyses the significance of redox equilibrium in the context of psoriasis. Based on information available from global literature published in the last decade, the authors analyse the impact of exogenous antioxidants present in food products on the course of this chronic disease.

## 2. Systemic Oxidoreductive Balance

The production of ROS must remain under close control of the enzymatic and nonenzymatic antioxidative system. Should the system become inefficient and/or should excessive ROS production be observed, a cell may suffer from imbalance between pro- and antioxidative factors that is typically referred as oxidative stress [9]. ROS-induced oxidative stress triggers inflammatory response as well as NF-κB protein-dependent gene transcription for various inflammatory factors [27]. The key parameters describing oxidative stress include enzymes: superoxide dismutase (SOD), catalase (CAT), and glutathione peroxidase (GPX), as well as nonenzymatic antioxidants present in cells, primarily glutathione (GSH) [9]. SOD catalyses the reaction of superoxide anion radical (O_2_•^−^) dismutation, which leads to the formation of hydrogen peroxide (H_2_O_2_) and molecular oxygen (O_2_) as a result of the reactions of reduction and oxidation of metal ions contained in active SOD centres. In peroxisomes, hydrogen peroxide is subject to further enzymatic dismutation to water and oxygen with the participation of CAT [9]. GPX is a selenoenzyme catalysing the reduction of hydrogen peroxide by GSH. The reaction produces oxidised glutathione [28]. GSH, one of the main nonprotein thiols present in living organisms next to cysteine, not only serves the role of an intracellular redox buffer—the direct “scavenger” of ROS—but also constitutes a cosubstrate in the reactions of ROS inactivation and the detoxication of xenobiotics catalysed by GSH-dependent enzymes. The -SH group in glutathione is far more easily available for oxygen than the thiol groups in enzymes, hence GHS is capable of protecting bioactive protein [9,29]. Cysteine is a reductive factor as well as substrate in the biosynthesis of GHS and a precursor for metabolically reactive sulphonic sulphur. Cysteine residue present on the surface of proteins serves antioxidative purposes and can also undergo reversible oxidation to unstable sulphonic acids [30]. The concentration of advanced oxidation protein products (AOPP) is considered to be a permanent marker of in vivo oxidative protein damage not only in the context of various inflammatory conditions accompanied by oxidative stress but also in the course of many diseases [31]. In order to ensure the correct level of GHS, it is necessary to maintain the adequate concentration of reductive equivalents. Reduced form of nicotinamide adenine dinucleotide phosphate (NADPH) is the most important intracellular reductant produced in the pentose pathway in cellular cytosol [32]. The enzyme responsible for maintaining the correct concentration of NADPH is glucose-6-phosphate dehydrogenase (G6PDH) which participates in the pentose pathway. Together with glutathione reductase, G6PDH keeps GSH in the reduced form, thus protecting the organism against oxidative stress [33]. As such, the role of G6PDH is primarily protective in this context. G6PDH is in fact the only source of NADPH in red blood cells, which renders them more susceptible than other cells to oxidative stress damage due to oxidation of thiol groups in haemoglobin and molecular membranes [33].

## 3. The Organism’s Antioxidative Status in Psoriasis

### 3.1. Blood and Serum

#### 3.1.1. SOD, CAT and GPX Activity

Kadam et al. [34] reported a reduction of the antioxidative status in the blood serum of patients suffering from psoriasis (*n* = 90) compared to healthy subjects (*n* = 30), which resulted in reduced activity of SOD and CAT (Table 1). The study also revealed increased levels of nitric oxide (NO) in psoriasis patients, which, in the authors’ opinion, may be due to the immunological response and inflammatory processes inherent in the aetiopathogenesis of this disease. Moreover, the less-than-optimum ratio of endogenic oxidants and antioxidants was observed to exacerbate the progress of psoriatic lesions. Kute et al. [35] observed lower SOD activity and higher malondialdehyde (MDA) levels in patients suffering from psoriasis as compared to healthy persons. Additionally, the authors emphasised that smoking and drinking alcohol can lead to further disturbance of systemic antioxidative balance, while alcohol consumption also enhances histamine synthesis, which also negatively affects skin condition. Wójcik et al. [36] studied the status of antioxidative equilibrium in the lymphocytes of patients suffering from regular psoriasis and psoriatic arthritis. The obtained results revealed an increase in prooxidative parameters (elevated ROS level, decreased CAT activity and lower GPX as compared to healthy subjects). In the cited study, the authors also observed elevated levels of transcription factor Nrf2 activators in lymphocytes. The authors concluded that differences in terms of the Nrf2 pathway may be responsible for the disturbed redox equilibrium in the lymphocytes of psoriasis patients. Lymphocytes are among the most important cells involved in the pathogenesis of the disease [36].

#### 3.1.2. Antioxidant Vitamins

The levels of blood serum vitamin E in erythrocytes are lower in psoriasis patients as compared to healthy subjects [37]. Johnson et al. [54] suggested that the pathway of vitamin A synthesis may be altered in psoriasis patients, which they inferred from the elevated (*p* < 0.05) levels of vitamin A and α-carotene in the blood serum of the affected subjects (*n* = 156) as compared to healthy individuals (*n* = 6104). On the other hand, contrary results were reported by Alwasiti et al. [39] who observed decreased levels of vitamin A in the blood serum of psoriasis patients as compared to healthy subjects.

#### 3.1.3. Thiol/Disulphide Balance

–SH groups, as constituents of compounds with antioxidative properties, are oxidised to –S–S– disulphide bridges, which reflects the loss of the compensation capacity of antioxidative mechanisms [29]. Studies conducted in a group of 29 patients suffering from psoriasis revealed a higher level of -SS disulphide bridges in their blood, as compared to healthy subjects, while the general thiol levels remained the same [40]. The results indicate disturbance of the thiol/disulphide balance as a result of oxidative stress and inflammation. The cited authors also observed that the –SS level did not show a clear correlation with the advancement of psoriasis measured with PASI (Psoriasis Area and Severity Index). In a study by Aksoy and Kirmit [41] conducted in a group of 80 psoriasis patients, the results indicated that the thiol–disulphide equilibrium was maintained, but the overall thiol level was lower (*p* < 0.01) in psoriasis patients as compared to healthy individuals, whereas the -SS levels revealed no statistically significant differences. In a study by Kilic et al. [42] lower levels of -SS and higher levels of native plasma thiol were reported for psoriasis patients (*n* = 92) as compared to healthy subjects, which is indicative of antioxidative imbalance in the organism.

#### 3.1.4. Lipid Levels and Lipid Peroxidation Biomarkers

Fatty acids metabolism disorders are considered to be one of possible causes of psoriasis [55]. In the case of skin dryness caused by deficits or abnormalities in terms of fatty acids metabolism, epidermal keratinisation disorders (keratosis) are often observed. Prostaglandins are among the elements responsible for regulating said processes. Lipid peroxidation is a chain, free-radical process of lipid oxidation with the participation of ROS [55]. The continuous production of reactive carbonyl species (RCS), by way of lipid peroxidation in the processes of oxygen metabolism, means that they take part in cellular homeostasis. The intensified lipid peroxidation induced by free radicals activates the process of oxidative stress, leading to the breakdown of membrane polyunsaturated fatty acids (PUFA) and consequently structural modification of cellular membranes [9]. MDA is a product of lipid peroxidation characterised by the greatest biological significance, commonly used as a marker of oxidative stress. ROS-mediated lipid peroxidation is considered to be one of the main causes of cell damage in psoriasis [44].

Numerous authors have reported that elevated total cholesterol and triglyceride levels in the blood serum were observed in psoriasis patients as compared to healthy individuals (Table 1). Studies in a group of psoriasis patients also revealed disorders in terms of lipid metabolism in the blood [36]. Boda et al. [43] reported increased lipid peroxidation in erythrocytes (ESP) for psoriasis patients (*n* = 32) compared to the control (*n* = 34), which was generated by intensified ROS effects at the erythrocyte level. The results of MDA determinations conducted by Pawłowska et al. [25] and Kadam et al. [56] indicated significantly higher concentrations of this compound in both the plasma and erythrocytes of psoriasis patients, compared to healthy individuals. This could suggest that the excessive production and harmful effects of ROS as well as the process of lipid peroxidation play a significant role in the pathogenesis of psoriasis. The unaffected concentration of primary lipid peroxidation products reported in the cited study, accompanied by significant changes in the concentration of the secondary products of this process observed in psoriasis patients, suggests a significant severity of free radical-induced cell damage. Undoubtedly, the process of lipid peroxidation resulting from excessive ROS production and impairment of the antioxidative system is exacerbated in patients suffering from psoriasis, whose serum and erythrocytes show elevated content of MDA resulting from decreased β-carotene, GSH, and α-tocopherol levels as well as increased SOD activity in red blood cells [57]. Bacchetti et al. [58] conducted a study in a group of 23 psoriasis patients to determine whether it is possible to modulate the lipid profile and antioxidative status of the same with the use of NTF-α inhibitors. The study revealed no significant (*p* < 0.01) impact of the analysed factor on the patients’ lipid profile, but a significant decrease in the degree of lipid peroxidation (LPO) was observed as well as improvement in terms of antioxidative status parameters: total antioxidative capacity (TAC) and paraoxonase (PON). Barygina et al. [59] also demonstrated a significant impact of the anti-TNF-α therapy on the redox equilibrium of psoriasis patients, most likely related to normalisation of NADPH oxidase activity in white blood cells. Additionally, the authors concluded that NADPH oxidase may constitute a leading source of ROS overproduction in the white blood cells of psoriasis patients. Pietrzak et al. [45], in a study on psoriasis patients (*n* = 93), observed lipid metabolism irregularities and oxidative balance disorders that may be due to chronic inflammation. Particularly high levels of oxidised LDL (ox-LDL) were observed in the serum of psoriasis patients where they were over five times higher than the corresponding control levels. The cited authors did not test for epidermal ox-LDL levels, but a study by Tekin et al. [60] revealed significantly elevated ox-LDL and anti-ox-LDL concentrations in patients suffering from psoriasis, particularly in the top epidermal layer, whereas the skin of healthy persons contained neither ox-LDL nor anti-ox-LDL. A study by Nemati et al. [44] conducted in a group of 100 psoriasis patients revealed that elevated lipid and lipoprotein levels (particularly TC, LDL, Lp(a), and ApoB) as well as reduced levels of antioxidants (SOD, CAT, PON1) may cause accumulation of ox-LDL and ROS in psoriasis patients, which in turn may play an important role in the context of immunological inflammatory states causing skin cell damage or arterial atherosclerosis in psoriasis patients. Furthermore, in a study by Asha et al. [46], psoriasis patients showed elevated levels of MDA, a marker of lipid peroxidation and ox-LDL, with the corresponding decreased levels of endogenous antioxidants. Increased concentration of MDA in the blood serum, as compared to healthy individuals, was also reported in a study by Kiran and Deedi [47] where the authors additionally observed elevated levels of alanine aminotransferase (ALT) in psoriasis patients, which indicated hepatotoxicity attributable to either oxidative stress or the effects of medicines administrated in the treatment of psoriasis. Moreover, research results indicate that LDL oxidation and an increase in ROS levels, apart from being markers of inflammation, may also play a role in inducing atherosclerosis in psoriasis patients [46]. Protein modifications by the products of lipid peroxidation and MDA promoted the proapoptotic pathway in lymphocytes, which led to the increased expression of proapoptotic caspases [36].

### 3.2. Epidermal Cells

Interesting results were also obtained by Portugal-Cohen et al. [61]. Contrary to most published studies, the cited authors concluded that psoriasis-affected epidermal cells actually showed higher antioxidant total scavenging capacity (TSC) as well as higher content of uric acid when compared to unaffected epidermal cells. Nonpathological cells originating from psoriasis patients returned lower levels of TSC and uric acid than those of healthy individuals. In the opinion of the cited authors, the same may suggest increased scavenging rates of overproduced ROS in cells affected by the disease. In a study by Abdel-Mawla et al. [48], the levels of CAT in the blood serum and erythrocytes were significantly (*p* < 0.05) higher in patients suffering from psoriasis as compared to healthy control, which, in the authors’ opinion, suggests oxidative damage and imbalance of the organism’s antioxidative system. On the other hand, Metta et al. [49] did not observe statistically significant differences in terms of CAT and SOD content in the blood serum of psoriasis patients and healthy individuals. Short-term exposure to oxidative stress increased the activity of exogenous antioxidants, which indicated activation of defence mechanisms and cell adaptive response, however, under long-term exposure, cellular activity became clearly reduced [9]. Another possible explanation could be that with the progression of inflammation in tissues and organs, including the epidermis and dermis, one of the SOD enzymes, SOD3, is expressed, although that expression is reduced in skin affected by psoriasis [62]. The skin is the organism’s primary barrier against exogenous threats such as exposure to UV radiation, chemicals and pathogens, it therefore needs to have a suitable endogenous strategy for preventing inflammation. As the tissular distribution of SOD3 is closely related to its substrates and functional position of peroxide production, it likely naturally protects tissues that are particularly strongly exposed to ROS by, for example, deactivating NADPH oxidase.

### 3.3. Saliva

Skutnik-Radziszewska et al. [38] analysed the levels of antioxidants and oxidants in the saliva of psoriasis patients. The authors concluded that the subjects’ antioxidative balance in the saliva and erythrocytes, evaluated on the basis of salivary peroxidase activity (PX), CAT, GPX, AOPP, advanced glycation end products (AGEP), MDA, and total lipid hydroperoxide (LOOH), was disturbed. In the saliva, the same resulted in elevated concentrations of oxidised biomolecules as compared to healthy individuals, with the highest sensitivity observed for secretions from submandibular glands.

## 4. Exogenous Antioxidants in the Diet of Psoriasis Patients

### 4.1. Diet Structure

Diet is an important factor in the treatment of many dermatological conditions. High consumption of fresh fruit and vegetables tends to correlate with lower incidence of psoriasis, while vegetarian diets have been observed to facilitate overall improvement in psoriasis patients [63,64,65]. The same is most likely due to the related high intake of antioxidants, which boosts the organism’s antioxidative capacity and solidifies the related equilibrium. Persons following a vegetarian diet on a long-term basis have been observed to show higher levels of antioxidant compounds in the blood serum and better lipid rates [66], although consuming increased amounts of vegetables over only several weeks was found to have no significant impact on those parameters [67]. There is a significant positive correlation between the overall intake of antioxidants and the organism’s antioxidative capacity [68]. Moreover, a vegetarian diet contains lower amounts of arachidonic acid responsible for stimulating the emergence of inflammatory eicosanoids [69]. A Brazilian study conducted in a group of 44 psoriasis patients revealed positive effects of supplementation with n-3 fatty acids on the organism’s antioxidative status [70].

Interesting results were obtained by Solis et al. [71], who studied the dietary habits of 34 male psoriasis patients aged between 19 and 60 years. The authors observed that the consumption of fresh fruit and vegetables in this group of patients was too low, which suggested a reduced intake of exogenous antioxidants. The researchers analysed the intake of vitamin (A, C, E, B-complex) and minerals (manganese, zinc, selenium) in this group and concluded that, on average, it did not exceed 50% of the respective requirement. In a similar study conducted in Italy by Barrea et al. [72], psoriasis patients (*n* = 62) were found to consume fewer fruit and vegetables and more red and processed meat (arachidonic acid) than healthy individuals. A Brazilian study also suggested that psoriasis patients followed an unhealthy died that predisposed them for the emergence of chronic diseases related to obesity and exacerbation of psoriatic lesions [71,73]. In Japan, psoriasis patients were observed to consume more fish and shellfish, leguminous plants, green and yellow vegetables, sugar and sweeteners, vitamin B12 and vitamin D, as well as less meat than healthy subjects [74]. The cited authors also reported that affected patients ingested more β-carotene and vitamin A with their diet, which evidenced radically different dietary habits of said psoriasis patients as compared to those from other parts of the world. Moreover, the same study indicated that persons with high PASI tended to consume more sweets than other sick individuals. A study conducted in the USA by Afifi et al. [75] demonstrated, in a group of 1206 psoriasis patients, that such persons were generally willing (86%) to modify their dietary habits in the course of the treatment, the authors also observed improvement in terms of skin condition after eliminating alcohol (prooxidative effects) and increasing the intake of vegetables and vitamin D (antioxidants).

Wong et al. [65] described the case of a woman who, having unsuccessfully tried to pharmacologically treat psoriasis-related skin lesions for 14 years, switched to a diet based on fresh vegetables, with a radical reduction of meat consumption and complete elimination of fast food and sugar. Additionally, the patient used vitamin-rich diet supplements containing e.g., vitamin C. After only six months of following the diet, the psoriasis lesions disappeared completely, despite having been significantly exacerbated in the first month of the therapy. The effectiveness of vitamin C in psoriasis treatment was also confirmed in a study conducted in a group of patients undergoing UVB radiation treatment [76]. The blood serum of patients receiving 500 mg of vitamin C for 12 weeks was observed to show decreased MDA levels and increased levels of GSH and vitamin C.

Curcumin, a natural polyphenol and yellow pigment obtained from curcuma spice shows strong antioxidative, anti-inflammatory, and antibacterial properties, which makes it highly recommendable for psoriasis patients [77]. One of the challenges related to maximising the therapeutic potential of curcumin is its low bioavailability, limited solubility in water, and chemical instability [78]. It has been demonstrated that curcumin can be effectively used as supplementary treatment in regular psoriasis as it significantly reduces the serum levels of IL-22 [79]. Research results show that the use of curcumin facilitates the improvement of the total antioxidative status (TAS) and decrease of MDA levels in the blood serum of rats [80] as well as in human mesenchymal stem cells isolated from adipose tissue [81]. In a study by Chen et al. [82], a clear increase in the SOD, CAT, and GSH activity was observed as well as a reduction of MDA levels in psoriatic cells, both in isolated human HaCaT cells and in a BALB/c mice model, after the use of a mixture of natural plant extracts, including curcumin.

Unfortunately, the use of diet supplements with antioxidative properties is not always effective. This may be due to both the dosage of antioxidants and the duration of treatment—studies suggest that, in order to be effective, supplementation requires consistent and long-term administration of antioxidants [83]. Zinc (Zn) has not been observed to be effective in psoriasis treatment [64], despite the fact that it is known to reduce the production of free radicals in the organism due to its inhibition of the activity of NADPH oxidase, its presence in Cu,Zn-SOD, and facilitation of the stability of –SH groups in proteins [21]. However, the blood serum of persons suffering from psoriasis has been shown to contain significantly higher levels of both Zn and copper (Cu) as compared to healthy subjects [39]. Selenium (Se) levels in the blood serum of psoriasis patients are lower than those in healthy individuals [84]. Selenium can influence immunological response by affecting the expression of cytokines and their receptors or increasing the resistance of immune cells to oxidative stress. As an ingredient of selenocysteine, it is active in the redox centre, which reduces the risk of oxidative damage spreading to lipids, lipoproteins, and DNA [85]. In a study by Serwin et al. [86] conducted in a group of 37 psoriasis patients, it was demonstrated that selenium supplementation, safely dosed (at 200 μg Se daily in the form of selenomethionine) for four weeks, proved ineffective in patients suffering from psoriasis. At the same time, it has been demonstrated that selenium molecules can prevent in vivo release of proinflammatory cytokines induced by UVB by inhibiting mRNA in human keratinocytes [85]. Inconclusive results have also been reported in terms of using folic acid, a vitamin with strong antioxidative properties [87]. American researchers demonstrated that daily administration of folic acid dosed at 1–2 mg/day can trigger or exacerbate psoriatic lesions, whereas increasing the dosage by two to four times results in weakening PASI [88].

### 4.2. Food Containing Antioxidant Compounds

Phenolic compounds present in many plant products can reduce or inhibit free radicals by transferring hydrogen atoms from their hydroxyl groups. The mechanism of the reaction between a phenolic compound and a peroxide radical (ROO•) includes the coordinated transfer of a hydrogen cation from the phenol to the radical, thus creating a transitional state of the H-O bond with a single electron [9]. Given the strong antioxidative properties of polyphenols contained in some food products, it could be advisable to include them in the diet of psoriasis patients, even though the available literature does not evidence their beneficial effects in such cases. However, due to their conclusively demonstrated impact on the organism’s antioxidative status, one can reasonably expect them to also benefit the antioxidative system of psoriasis patients.

#### 4.2.1. Tea

The popularity of tea as a beverage is exceeded worldwide only by water. Due to its content of antioxidants, it meets the conditions for classification as functional food [89]. The antioxidative properties of tea are due to its high content of polyphenols, mainly catechins, including epigallocatechin-3-gallate (EGCG) present in green tea, quercetin, theaflavin, and thearubigin present in black tea, as well as tannic acid [9]. The particularly high antioxidative activity of EGCG stems from the presence in its structure of eight -OH groups [9]. The activity of catechins stems not only from the antioxidative processes of donating H, but most likely also from the mechanisms and pathways that directly or indirectly regulate the expression of enzymatic antioxidants [90]. The total polyphenol content in tea reaches 25–35% of leaf dry mass [91]. The highest content thereof (determined as tannic acid equivalent) is found in white tea (2668 mg/1000 mL), followed by green (2363 mg/1000 mL), black (1220 mg/1000 mL), and red varieties (996 mg/1000 mL) [83]. The increased activity of SOD, CAT, GST, and GPX observed in numerous studies, as well as elevated GSH content and reduced LPO and MDA levels in in the organs of animals and humans receiving tea solutions, indicates improvement of antioxidative mechanisms’ efficiency thanks to the supply of exogenous antioxidants, which facilitates maintaining the equilibrium of redox reactions and prevents the onset of oxidative stress [9]. It has been demonstrated that the antioxidative potential in the blood serum is increased by 34% after drinking green tea and by 29% after drinking black tea [92]. Notably, green tea is characterised by higher antioxidative activity than white or black tea, as confirmed in vitro in a study conducted on isolated human erythrocytes [93]. The increase of blood plasma antioxidative activity after drinking tea is dependent on the dosage [94]. Numerous studies conducted on laboratory animals confirmed the positive impact of tea on the antioxidative status of organisms poisoned with prooxidative substances [9]. Wistar rats poisoned with prooxidative toxic metals showed an increase in SOD, CAT, and GPX activity in their organs after 12 weeks of receiving black, green, white, and red tea infusions [83] as well as tannic acid [95]. After intraperitoneal administration of catechin and epicatechin to rats, a decrease in MDA levels in urine and increased SOD concentration was observed [90]. Similarly, administering polyphenols isolated from green tea to mice (50, 100, or 200 mg/kg) resulted in an improvement of antioxidative parameters (SOD, CAT, GPX, MDA, LPO) in their blood serum [96]. The GPX reduction observed in the cited study (without an impact on TAC) may be due to the fact that catechins protect GSH (which is a substrate of GPX) against free radicals [97].

#### 4.2.2. Coffee

Similarly to tea, coffee is among the most popular beverages worldwide. It is a source of antioxidants—research results suggest that drinking coffee may increase the level of GSH and facilitate protection against DNA damage, especially if consumed regularly [98]. The antioxidative activity of coffee is due to the presence of phenolic acids: chlorogenic, ferulic, caffeic, and n-coumaric, as well as caffeine and trigonelline [99]. In the process of roasting coffee beans, melanoidins (brown pigments) are synthesised which show strong antioxidative properties [99]. Phenylalanines and heterocyclic compounds formed in the roasting process also show strong antioxidant activity [100]. The total content of polyphenols in coffee is higher than in tea, with green coffee exceeding roast coffee in this respect [101,102]. Moreover, instant coffee contains more total polyphenols compared to the grain variety, it also shows higher antioxidant activity [103]. Nonetheless, the results of studies conducted on humans remain inconclusive, some indicate improvement in terms of antioxidative parameters, others an increase in MDA content, and yet others absence of any correlation whatsoever between drinking coffee and the levels of antioxidative parameters in blood serum or saliva [98,104,105]. Jung et al. [106] argue that coffee shows physiological antioxidative and anti-inflammatory properties and its effects are negatively correlated with the levels of grain roasting (light, medium, city, and French), which was demonstrated in experiments conducted on cellular models.

#### 4.2.3. Herbs and Spices

Some herbs used in the kitchen or for therapeutic or treatment purposes also contain antioxidants. In the case of common marigold (*Calendula officinalis* L.) these include in particular flavonoids (flavonols and flavone glycosides), coumarins, sterols, and carotenoids [107]. St John’s wort (*Hypericum perforatum* L.) contains flavonoids and phenolic acids, and in oregano (*Origanum vulgare*) we find a range of phenolic compounds including protocatechuic, caffeic, coumaric, and rosmarinic acid, as well as quercetin [107]. A study conducted on laying hens revealed that administration of flavonoids, including quercetin, statistically significantly reduced their MDA and increased SOD, GSH, and GST levels [108]. Similarly, administering water oregano solutions to rats resulted in a significant (*p* < 0.05) reduction of MDA and increase of CAT and SOD in the kidneys compared to animals not receiving the extracts; similar results were also reported for quercetin [109]. The addition of oregano oil to the fodder mixture consumed by pregnant and farrowing sows reduced the systemic oxidative stress observed during pregnancy [110]. Oregano oil was also fed to sheep, which led to a general improvement of oxidative stress parameters in their meat and livers [111].

The addition of thyme oil to the diet of Japanese quails resulted in an increase of antioxidant parameters (SOD, CAT, GSH) and reduction of MDA in the blood serum and livers, compared to birds receiving standard diet [112]. Similar results were obtained in other animals with the use of various forms of thyme: rats [113], broilers [114], and fish [115]. The strong antioxidative properties of thyme are due to its content of phenolic compounds, particularly carvacrol and thymol, as well as monoterpenes (e.g., p-cymene, α-pinene, β-pinene, α-terpinene, limonene, myrcene, terpene alcohols) [113,116,117]. The antioxidative effectiveness of thymol was demonstrated in a study on rats poisoned with CCL_4_ [118]. Similar effects were reported when thymol and carvacrol were used in an in vivo study on human hepatic cancer cell lines HepG2 subjected to a strong dose of acetaminophen (APAP) [119].

Dill (*Anethum graveolens* L.) contains numerous volatile ingredients with antioxidative properties, e.g., 7-α-hydroxy-manool, l-carvone, limonene, epi-α-bisabolol, α-terpinene, and α-phellandrene [120]. In the cited study, the use of water dill extract (3 g 100 mL^−1^ pf water) for 30 days in rats receiving prooxidative paracetamol yielded an improvement in terms of parameters describing oxidative balance: TAC, CAT, and GPX. Thanks to its content of polyphenols and antioxidative vitamins, cistus (*Cistus incanus* L.) lowers MDA and AOPP levels in the blood serum of people drinking the infusion as early as after 6 weeks of use [121].

The use of water or alcohol cinnamon extract improved oxidative stress parameters (↓ MDA, ↑ SOD, ↑ CAT) in the livers of rats poisoned with prooxidative tetrachloromethane (CCl_4_) [122]. Similarly, in another study conducted on rats, oral administration of a water solution of powdered cinnamon (75 mg/kg/day) resulted in an increase in the serum SOD, CAT, and GPX levels and decrease of MDA [123]. In rats receiving a high-fat diet, the inclusion of cinnamon lowered MDA and increased TAC, SOD, CAT, and GPX levels in the blood serum and/or liver, while at the same time no influence of cinnamon on the oxidative status of the control group animals was observed [124]. After 12 weeks of administering capsules containing a water extract of cinnamon to patients suffering from glucose metabolism disorders, an increase in the number of –SH groups and oxidative status was observed, as well as lowered plasma MDA levels [125]. However, in the cited study no impact of cinnamon on the activity of SOD or CAT in red blood cells was reported. The authors attributed the observed oxidative stress reduction to the content of antioxidative phenolic compounds in cinnamon. The most potent antioxidants present in cinnamon bark include polyphenols (flavonoids and tannins) as well as volatile phenolic oils [126]. Moreover, the cited authors mentioned that the highest antioxidant activity was observed for traditionally prepared cinnamon solutions. Cinnamon contains over 30 times the amount of total phenols and approximately three times the amount of polyphenols compared to, for example, garlic or curcuma [127]. Hence, its antioxidant activity, i.e., the ability to scavenge free radicals, is very high [128].

Garlic extract (*Allium sativum*) administered to rabbits poisoned with prooxidative carbon tetrachloride CCl_4_ stimulated improvement of hepatic antioxidative parameters: MDA, GSH, CAT, SOD, as well as lipid parameters [129]. Also studies conducted on rats confirmed an improvement in terms of TAS levels after oral administration of garlic extract (600 mg kg^−1^) for one month, as compared to control animals receiving distilled water [130]. A water extract of garlic can contain up to 129 mg of phenolic compounds in 1 g, including 101 mg of flavonoids and 94 mg of flavonols, which translates to very high antioxidative potential [131]. It has been shown that aged garlic obtained in the process of fermentation is characterised by even better antioxidative properties than its fresh counterpart and, as such, meets the criteria for classification as functional food [131]. Studies conducted on rats and mice with a water extract of aged garlic corroborated its beneficial effects on tissular redox status parameters (MDA, GPX, GSH, SOD, CAT, TBARs, –SH groups) [132,133]. During garlic fermentation, allicin is transformed into water-soluble antioxidants such as S-allyl-cysteine, tetrahydro-β-carbolines, and bioactive alkaloids. S-allyl-cysteine is the most important bioactive compound found in garlic and its content in aged garlic exceeds that found in the fresh variety [131].

#### 4.2.4. Fruit

Many types of fruit provide valuable sources of phenolic compounds, primarily flavonoids (anthocyanins, flavanols, quercetin, rutin, resveratrol), condensed tannins (proanthocyanidins), tannins (ellagitannins, gallotannins, tannic acid), stilbenes, hydroxybenzoic acids (ellagic and gallic) and phenolic (caffeic, chlorogenic, ferulic), as well as well as antioxidative vitamins [134,135,136,137]. The most valuable types of fruit include berries (blueberries, blackberries, raspberries, cranberries, strawberries, grapes, and others) as well as plums, pomegranates, apples, and grapefruits [134]. Research results confirm that extracts from various berry varieties show antioxidant activity, e.g., the ability to scavenge hydroxyl radicals, singlet oxygen, hydrogen peroxide and superoxide radicals, inhibit lipid peroxidation and ROS generation, as well as boost thiol levels and the activity of antioxidative enzymes [134,138]. Consuming a mixture of various berries over a period of eight weeks resulted in an increase in the blood serum vitamin C level compared to the control, but had no significant impact on the overall oxidative capacity [139], whereas after two weeks of consuming 300 mL of antioxidant-rich red grape and lingonberry juice (80:20), researchers observed increased SOD and CAT levels and lowered blood serum MDA as compared to the control group [140]. Studies conducted on rats revealed that oral administration of dried ethanol plum extract (150 mg kg^−1^ body weight) improved the oxidative stress parameters (H_2_O_2_, MDA, GSH, SOD, CAT) in the bones compared to animals poisoned with dexamethasone, but had no impact on said parameters when compared to control group animals [141]. The use of a methanolic extract of polyphenols obtained from apples on adenomatous human stomach cells MKN 28 prevented oxidative damage to those cells, triggered a fourfold increase in intracellular antioxidative activity, and prevented subsequent decrease thereof and lipid peroxidation induced by xanthine oxidase [142]. The main phenolic constituents include catechins and chlorogenic acid [142]. Intraperitoneal injection of 1 mg resveratrol, a flavonoid present, e.g., in grape skin, to rats suffering from spinal cord ischaemia, elevated the levels of enzymatic and nonenzymatic antioxidants such as GSH, SOD and CAT, and decreased MDA [136]. Also the consumption of pomegranate juice rich in polyphenols has been found to improve antioxidative parameters (CAT, GPX, MDA) in the blood serum of persons practicing strength sports [143]. Studies conducted on rats receiving methotrexate, a prooxidative drug used in the treatment of psoriasis, showed an improvement in terms of antioxidative parameters (GPX, SOD, MDA) and the serum lipid profile (TC, HDL-chol, LDL-chol) after the oral administration of 500 mg per kg of pomegranate in the form of a methanol extract from seeds or peels [144,145]. All the parts of a pomegranate (peel, seeds, arils) contain polyphenols, particularly flavonols, flavonoids, gallic acid, quercetin, tannins and anthocyanins, as well as vitamins C and E and β-carotene, which means that the fruit is characterised by particularly strong antioxidative activity [146,147].

## 5. External Application of Antioxidants in Psoriasis

Flavonoids are natural compounds with strong anti-inflammatory and antioxidative properties. Luteolin inhibits TNF-induced phosphorylation, nuclear translocation and DNA binding the nuclear factor kappa B (NF-κB) usually involved in the transcription of an inflammation mediator [148]. It was also observed that luteolin reduces the TNF-induced mRNA expression of two genes (NFKB1 and RELA) coding two NF-κB subunits (respectively NF-κB p50 and NF-κB p65). Interestingly, the expression of RELA genes is elevated in psoriatic skin. Studies have revealed that in human psoriatic HaCaT cells, astilbin—a glycoside flavonoid isolated from the root of *Smilax glabra*, a plant used in traditional Chinese medicine in the treatment of autoimmunological diseases, can induce translocation of the Nrf2 nucleus, which contributes to the reduction of ROS accumulation, expression of the vascular endothelial growth factor (VEGF), and inhibition of HaCaT proliferation stimulated by IL-17 and TNF-α [149]. The cited authors demonstrated that positive effects could be obtained with as little as 50 μg of astilbin. The use of antioxidative balsacones isolated from balsam poplar (*Populus balsamifera* L) on human fibroblasts harvested from fragments of psoriatic skin reduced the cell growth rate and stabilised the expression of cells in involucrin and loricrin more effectively than methotrexate [150]. Local application of propolis, due to its content of flavonoids, helps to improve the condition of psoriatic skin by suppressing the activity of macrophages and production of ROS [151]. Positive results were also observed after applying resveratrol on psoriatic cells [152]. Local administration of EGCG in BALB/c mice with psoriatic lesions induced with the use of imiquimod led to increased SOD and CAT activity in the plasma, as well as lowered MDA, as compared to the control group mice [153]. Moreover, the treatment also resulted in reducing the extent of overall disease-related skin lesions by limiting the expression of the epidermal proliferating cell nuclear antigen (PCNA), alleviating dermatitis accompanied by reduced infiltration of T cells, and lowering the levels of proinflammatory interleukins IL-17A, IL-17F, IL-22, and IL-23. Positive effects have also been reported after external application of caffeine on psoriatic skin [154]. Caffeine metabolites show antioxidant properties, which helps to reduce the inflammation caused by lipid peroxidation, DNA damage, and damage to cellular organelle.

## 6. Clinical Studies

The use of exogenous antioxidants results in a lower likelihood of inducing oxidative damage due to the decreased antioxidative capacity of the organism’s defence system. The activity of exogenous antioxidants can be threefold: (1) synergistic—i.e., scavenging oxygen and chelating compounds catalysing oxidation reactions; their activity entails donating hydrogen to phenoxy-radicals, which restores their original antioxidative properties, (2) disruption of radical reactions by donating hydrogen atoms or electrons, which stabilises the form of the radical, as well as (3) mixed reactions, such as electron transfer coupled with proton transfer, electron transfer preceded by deprotonation, or proton transfer preceded by electron transfer [21].

Clinical studies conducted on psoriasis patients revealed that antioxidative strategies may constitute viable therapeutic methods complementing pharmacological treatment. Cellular signal pathways such as protein kinase, κB nuclear factor, or Janus kinase signal transducers are susceptible to oxygenation, and it has been demonstrated that they contribute to the progression of psoriasis [2]. Since 1959, drugs containing dimethyl fumarate (DMF) have been used orally in the treatment of various stages of psoriasis with considerable success [2]. DMF acts by chemically modifying the repressor protein Keap 1 that allows stabilisation and transfer to the cell nucleus of the transcription factor Nrf2, which in turn results in the activation of a cascade of several pathways with cytoprotective and antioxidative properties [155]. Keap1 is among the most important intracellular markers of the redox state, sensitive to inorganic and organic hydroxides, peroxynitrites, and other electrophilic molecules forming under the conditions of oxidative stress and tissue damage [156]. The results of in vitro studies and experiments on animal models indicate that DMF mediates the increase of glutathione levels and NADPH induction [2].

Studies conducted on psoriasis patients corroborated the effectiveness of exogenous antioxidants as a supplementary therapy in the course of pharmacological psoriasis treatment. Madhulatha and Vijayabhaskar [157] demonstrated a significant decrease of MDA and increased total antioxidative capacity (TAC) in the blood serum of patients receiving pharmacological treatment already after eight weeks of therapy using a mixture of antioxidants (30 mg β-carotene + 27.5 mg zinc sulphate monohydrate + 0.2 mg monohydrated selenium dioxide + 2 mg manganese + 1 mg copper). In a study by Kharaeva at al. [158], 58 patients suffering from severe psoriasis or psoriatic arthritis underwent supplementary treatment entailing oral administration of a mixture of oxidants: Q10 coenzymes, vitamin E, and selenium. After a month of therapy, normalisation was observed in terms of CAT and SOD activity in granulocytes and epidermal cells affected by psoriasis, as compared to the control group of psoriasis patients receiving placebo. Also in a study conducted on isolated Colo-16 cells exposed to UVB radiation, a decrease in ROS levels and increased activity of SOD and CAT was observed after the use of vitamin C and E and Ginsenoside Panaxatriol [159]. At the same time, in a study by Abdel-Mawla et al. [48], a therapy using a mixture of antioxidants (100 μg selenium + 1500 IU vitamin A + 90 mg vitamin C + 30 mg vitamin E) administered orally in the form of pills to psoriasis patients proved ineffective when compared to patients not receiving antioxidants as it failed to improve their antioxidative parameters (MDA, SOD, CAT) and skin condition. The latter results are difficult to explain given the fact that numerous authors have reported improvement of the antioxidative parameters in psoriasis patients even when using considerably lower doses of antioxidants. For instance, Kharaeva et al. [158] administered a mixture of antioxidants containing 50 mg of vitamin E, 48 µg of selenium, and 50 mg of coenzyme Q10 to their patients and observed improvement of antioxidative parameters after 30–50 days of therapy (Table 2).

A study conducted in a group of 28 psoriasis patients revealed that methotrexate, an organic compound belonging to the group of folic acid antagonists and used as a cytostatic drug (thanks to the inhibition of the NF-κB transcription factor whose excessive transcription can lead to increased production of IL-6 and IL-8 interleukins in the skin), apart from slowing down the multiplication of quickly dividing cells, is also capable of improving oxidative stress parameters (↓ MDA, ↑ total antioxidant capacity TAC) as compared to patients not receiving the drug [43]. It was additionally observed that the use of methotrexate alleviates dermal symptoms of psoriasis measured with the PASI scale. In turn, Barygina et al. [161] concluded that low doses (10 fg per mL) of IL-4, IL-10, and IL-11 cytokines administered orally reduce oxidative stress in the primary fibroblasts of skin fragments affected by psoriasis. Dermal fibroblasts provide a vital microenvironment for the epidermal function of keratocytes, and upon infiltration of polymorphonuclear leukocytes they start to intensively produce peroxide and H_2_O_2_, disrupting the redox equilibrium in psoriatic skin. In a clinical study, the cited authors concluded that oral administration of cytokines also resulted in a significant reduction of the PASI index compared to psoriasis patients not receiving cytokines. Cytokines influence the growth, proliferation, and excitation of cells, apart from being involved in the organism’s immune response, they also partake in pathological processes and show cytotoxic effects [162]. Moreover, some cytokines not only show antioxidative properties [43] but indeed their expression is affected by the cellular redox status [61], which is why it is vital to stabilise their levels in psoriasis patients. Interleukins IL-12 and IL-23 have been discovered to play a vital role in the pathogenesis of psoriasis through their participation in the interaction between immune system cells and proinflammatory cytokines [163]. They stimulate the differentiation of T lymphocytes into the subpopulation of Th1 and Th17, and subsequently their proliferation, which constitutes a source of other inflammatory mediators, e.g., TNF-α, IL-6, and IFN-γ, although currently, the greatest focus is on IL-17 and IL-22 [160].

A study conducted in a group of 60 psoriasis patients revealed that a 12-week treatment with the seeds of *Nigella sativa* in the form of ointment containing oil and/or small capsules taken orally resulted in a decrease in blood serum MDA and partial alleviation of skin lesions compared to patients receiving standard methotrexate treatment [164]. In the cited study, particularly spectacular effects were reported for the simultaneous use of the ointment and capsules. Also a study conducted on a rabbit model yielded increased GSH levels and lowered MDA in the blood serum and liver after 39 days of using a paste containing powdered *Nigella sativa* seeds, as compared to rabbits receiving methotrexate [165]. Nigella also shows antioxidative properties that exceed those of ascorbic acid and α-tocopherol [166,167], and its consumption facilities a better antioxidative capacity of the organism. The above properties stem from the content of numerous phenolic compounds [56].

The use of diatomic hydrogen molecules (H_2_) has been found to be safe and effective due to their antioxidative and immunomodulating properties with negligible side effects [168,169]. Unlike other antioxidants unable to target specific organelles, H_2_ can penetrate biomembranes and enter the cytosol, mitochondria and cell nuclei [168]. It has been proven to selectively eliminate ROS and show a positive impact on cytokine balance, particularly proinflammatory Th1 and Th2 [170]. H_2_ inhibits the chain reaction producing lipid peroxides and generating oxidative stress markers related to exacerbation of psoriasis symptoms. Moreover, the peroxynitrite formed in the reaction between nitrogen oxide and peroxide activated the p38 MAPK pathways related to the production of inflammatory cytokines such as TNF-α, IL-6 and IL-8, which stimulates the development of dermal psoriatic lesions [170]. The available research results pertain to hydrogen water (solubilised H_2_) administered orally and externally, as well as effective inhalation of hydrogen gas and injections of normal saline with H_2_ [169]. Zhu et al. [170] put 41 psoriasis patients through a treatment supplementary to the standard therapy and entailing baths in hydrogen water, which yielded positive results as measured in terms of PASI. A study conducted in a group of three psoriasis patients with the use of H_2_ in the form of saline injections (containing 1 ppm of H_2_), 3% H_2_ inhalations, and drinking water containing a high concentration of H_2_ (5–7 ppm) corroborated the high effectiveness of the treatment employed, as assessed on the basis of PASI and inflammatory cytokine levels [19]. Of the three methods of H_2_ administration, inhalation proved particularly effective, which may be due to the fact that H_2_ is absorbed into arterial blood in the lungs, which allows it to circulate in the organism and be ultimately released through the skin. The cited authors concluded that the beneficial effects were due to the high antioxidative activity of H_2_, as evidenced by the lowered serum levels of proinflammatory cytokines playing a key role in the pathogenesis of psoriasis, as compared to the pretreatment levels of the same. This suggests the participation of internally produced free radicals in the progression of psoriatic lesions, as well as the therapeutic potential of H_2_. Administering H_2_ reduces the expression of oxidative stress markers such as myeloperoxidase (MPO), MDA, 8-hydroxy-deoxyguanosine 8-OHdG, 8-iso-prostaglandin F2a and substances reactive with thiobarbituric acid in humans and animal models [171].

## 7. Perspectives

What can be inferred from the available literature is that the main research focus ought to be on effectively combining pharmacological therapy with the use of exogenous antioxidants in the treatment of psoriasis patients or persons predisposed for this disease. Nutrigenomics seems promising in this context as it guarantees a multiaspect approach to the question at hand. The focus of nutrigenomics is on the impact of food ingredients on the expression of genes, i.e., the functioning of cells [172]. Nutrients act as ligands for the receptors of transcription factors, serve as signalling particles and are metabolised in order to modify the concentrations of substrates or intermediates formed in the altered phenotype. Nutrigenomics is still a young science and its concepts are not easy to implement given the difficulty inherent in corroborating interaction between a given gene and a given nutrient in the course of clinical studies, primarily due to the chemical complexity of food and genetic diversity of humans resulting in different physiological response to particular food ingredients. Nonetheless, it has been demonstrated that dietary factors can affect the level of DNA methylation in humans [173]. Antioxidant-rich diets facilitate DNA demethylation by repairing enzymes, which was evidenced in an experiment where volunteers received a diet containing 300 g of vegetables and 25 mL of chestnut oil for a period of eight weeks [174]. Environmental and dietary factors, particularly isolated nutrients or bioactive compounds, can also control microRNA. It is a group of small, endogenous, single-strand, noncoding RNA with the length of 20–22 nucleotides, partaking in the regulation of gene expression at the post-transcription level by way of epigenetic changes, and identified as regulators of many metabolic processes [175]. MicroRNA molecules are usually involved in post-transcriptional gene silencing by inducing mRNA degradation or translational repression through binding with target informational RNA [176]. It has been demonstrated that they are involved in skin morphogenesis. The therapeutic effects of microRNA inhibition or overexpression have been observed on mice models [177]. Given the role thereof as the main triggers of complex cellular processes, they may potentially serve therapeutic purposes in inflammatory skin conditions.

Hawkes et al. [175] demonstrated incorrect expression in over 250 microRNA isolated from psoriasis-affected keratinocytes, adjacent healthy cells, and peripheral blood of patients. In the course of psoriasis, the balance between the respective types of microRNA is upset due to incorrect expression, which suggests that it may indeed constitute one of the primary causes of the disease [178,179]. It has also been observed that inflammatory processes and disruptions of the redox equilibrium stem from incorrect expression of microRNA, which suggests the influence of some substances present in food on the endogenic synthesis of microRNA under experimental conditions, both in vivo and in vitro. Numerous data corroborate the beneficial impact of antioxidants on the microRNA equilibrium. For instance, quercetin, a flavonoid present in fruit, effectively elevates the level of MiR-146a, which leads to a reduction of proinflammatory NF-κB and decreased expression of TNF-α, IL-6, and IL-17 [180,181]. In turn, vitamin D and quercetin modulate miR-125b, which regulates the transcription of NF-κB and expression of proinflammatory cytokines [178]. Studies conducted on mice receiving, for six weeks, a diet rich in quercetin dosed at 0.1 mg g^−1^ of feed, revealed reduced expression of proinflammatory miR-155, which resulted in inhibition of NF-κB activation [182]. Resveratrol, vitamin D, and curcumin cause a decrease in miR-21 and miR-155, which regulates anti-inflammatory mechanisms by reducing the production of proinflammatory cytokines, such as TNF-α, IL-1β, IL-6, and IL-8 [178,183,184]. In a study with the use of human monocytes, Tili et al. [185] demonstrated that resveratrol increased the level of anti-inflammatory miR-663 and lowered proinflammatory miR-155, which affected the expression of cytokines. Similar results with respect to proinflammatory miR-21 in human glioma cells were reported by Li et al. [183]. Hydroxytyrosol is a phenolic compound present in olive oil which has proven antioxidative properties. Its direct application on macrophages results in the reduction of proinflammatory miR-146a levels therein [186].

Diet manipulation may be a promising therapeutic approach in modulating the risk of chronic diseases. Therefore, it is advisable for psoriasis patients and persons predisposed for the disease to consume functional food, primarily containing high quantities of antioxidants. However, for this potential to be fulfilled, further consistent and intensive scientific scrutiny will be required.

## 8. Conclusions

**Figure 1:**
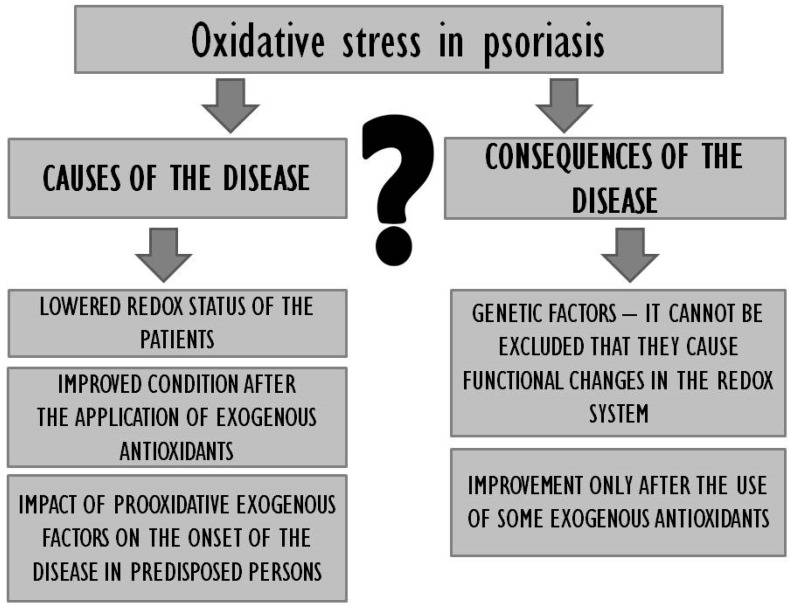
Oxidative stress in psoriasis—causes or consequences of the disease.

One should first consider whether oxidative stress is a cause or effect of psoriasis (Figure 1). The emergence of disease symptoms under conditions of oxidative stress as well as the clear improvement of the redox status in patients receiving exogenous antioxidants as well as the observable improvement of their general condition seem to suggest that oxidative stress may indeed be a cause of psoriasis.

**Figure 2:**
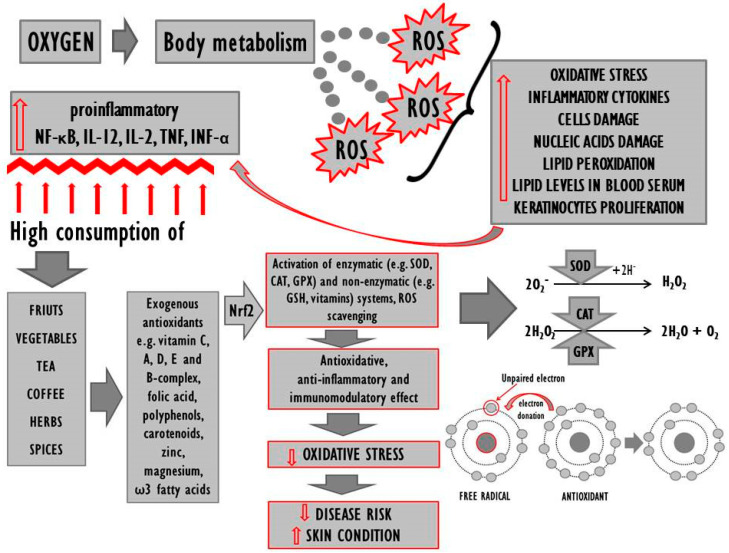
The effects of dietary supplementation with antioxidants in patients with psoriasis. Legends: ROS—reactive oxygen species; SOD—superoxide dismutase; CAT–catalase; GPX—glutathione peroxidase; GSH–glutathione; NF-κB—nuclear factor kappa B; IL–interleukins; TNF—tumour necrosis factor; IFN-α—alpha interferon; Nrf2—nuclear factor erythroid 2-related factor 2.

On the other hand, however, the fact that only some exogenous antioxidants prove effective despite the improvement in terms of the redox status as such would indicate that oxidative stress may be a consequence of the disease. Moreover, the available literature was found to lack data with regard to the redox status of persons genetically predisposed for but not suffering from psoriasis, it is therefore unclear whether the antioxidant levels in people whose genome includes genes responsible for the onset of the disease are imbalanced, and therefore whether the genes as such trigger functional changes in the redox system. In order to inactivate free radicals and stabilise cellular membranes, as well as to prevent further damage to the epidermis, one should consider the prophylactic consumption of antioxidants by genetically burdened persons and using the same to complement traditional therapy if the disease occurs. It is also worth considering certain lifestyle changes in the case of psoriasis patients, as research suggests that unhygienic habits (drug use, smoking, alcohol use) tend to increase the production of ROS in the organism and exacerbate the symptoms of psoriasis [35]. It is noteworthy that some ROS act only as signal messengers regulating a wide spectrum of physiological processes [187], which means that the perfect antioxidant ought to alleviate excessive oxidative stress but, at the same time, not interfere with redox homeostasis. Effective improvement of antioxidative parameters tends to be more viable with the use of a mixture of antioxidants as their respective effects will complement each other. It is necessary to develop a combination of pharmacological treatment and dietary antioxidant supplementation in psoriasis patients that would yield optimum results in the context of treatment and prevention. The results of numerous studies reveal that regular consumption of antioxidants in the diet can improve the organism’s antioxidative capacity and stabilise the related equilibrium. High consumption of fresh fruit and vegetables correlates with lower incidence of the disease [63,64,65]. The diet of people suffering from or genetically predisposed for psoriasis should include antioxidative n-3 fatty acids, vitamins C, A, D, E, and B-complex, with a simultaneous minimization of sweets and alcohol consumption (Figure 2). They should be encouraged to follow a diet rich in polyphenolic compounds such as tea, coffee and a variety of herbs and spices. Unfortunately, the use of diet supplements with antioxidative properties does not always prove effective, which may be due to the dosage of antioxidants and the overall duration of treatment. Promising results have also been reported in the context of nutrigenomics, which guarantees a multiaspect approach to the discussed problem. Future directions may entail detailed studies of psoriasis patients’ genetic profiles to allow more accurate targeting of therapeutic solutions.

## Figures and Tables

**Table 1:** Plasma antioxidant capacity and lipid peroxidation biomarkers in psoriasis.

Characteristic	Antioxidant Capacity	Lipid Levels	Peroxidation Biomarkers	References
Psoriasis *n* = 26 Control *n* = 16	≈ SOD		↑ MDA	[25]
Psoriasis *n* = 90 Control *n* = 30	↓ SOD; ↓ CAT; ↑ NO; ↓ TAS		↑ MDA	[34]
Psoriasis *n* = 90 Control *n* = 90	↓ vitamin E; ↓ CAT		↑ MDA	[37]
Psoriasis *n* = 50 Control *n* = 50	↓ SOD; ↓ GPX		↑ MDA	[35]
Psoriasis *n* = 40 Control *n* = 40		↑ TG; ↑ TC	↑ MDA	[38]
Psoriasis *n* = 48 Control *n* = 16	↑ NOX; ↑ ROS; ↓ CAT; ↓ GSH; ↓ vitamin E; ↓ GPX		↑ MDA	[36]
Psoriasis *n* = 50 Control *n* = 50	↓ SOD; ↓ vitamin E; ↓ vitamin A; ↓ Zn; ↓ Cu		↑ MDA	[39]
Psoriasis *n* = 29 Control *n* = 30	≈ native SH; ≈ total SH; ↑ SS; ≈ SS/total SH			[40]
Psoriasis *n* = 80 Control *n* = 80	≈ native SH; ↓ total SH; ≈ SS; ↑ SS/total SH			[41]
Psoriasis *n* = 92 Control *n* = 71	≈ native SH; ≈ total SH; ↑ SS; ↓ SS/total SH	↑ TC; ↑ TG; ≈ HDL-chol; ≈ LDL-chol		[42]
Psoriasis *n* = 32 Control *n* = 34	↓ G6PDH		↑ ESP	[43]
Psoriasis *n* = 100 Control *n* = 100	↓ SOD; ↓ CAT	↑ TC; ↑ LDL-chol; ↓ HDL-chol; ↑ ApoB; ↑ Lp(a); ↑ TG	↑ MDA; ↓ PON	[44]
Psoriasis *n* = 93 Control *n* = 60		↓ HDL-chol; ≈ LDL-chol; ≈ TG; ≈ TC; ↓ ox-LDL		[45]
Psoriasis *n* = 150 Control *n* = 150	↓ RGSH	↓ HDL-chol; ≈ LDL-chol; ↑ TG; ↑ TC; ↑ ox-LDL;	↑ MDA	[46]
Psoriasis *n* = 50 Control *n* = 50			↑ MDA	[47]
Psoriasis *n* = 34 Control *n* = 30	↓ SOD; ↑ CAT		↑ MDA	[48]
Psoriasis *n* = 60 Control *n* = 60	↓ GPX; ≈ CAT; ≈ SOD	↑ TG; ↑ TC; ↑ LDH-chol; ≈ HDL-chol	↑ MDA	[49]
Psoriasis *n* = 30 Control *n* = 30	↓ SOD; ↓ GPX		↑ MDA	[50]
Psoriasis *n* = 70 Control *n* = 30		↑ TG; ↑ TC; ↑ LDH-chol; ↑ VLDL-chol; ≈ HDL-chol	↑ MDA	[51]
Psoriasis *n* = 23 Control *n* = 23	↑ NO		↑ MDA	[52]
Psoriasis *n* = 50 Control *n* = 50	↓ SOD; ↓ GPX	↑ TG; ↑ TC; ↑ LDH-chol; ↓ HDL-chol; ↑ VLDL-chol	↑ MDA	[53]

↑—Increased concentration or activity in comparison to the control (healthy) group; ↓—decreased or inhibited concentration or activity in comparison to the control (healthy) group; ≈ no differences in comparison to the control (healthy) group; SS—disulfides; SH—thiol; TC—total cholesterol; HDL-chol—high-density lipoprotein cholesterol; LDL-chol—low-density lipoprotein cholesterol; VLDL-chol—very low-density lipoprotein; G6PDH—glucose-6-phosphate dehydrogenase; ESP—lipid peroxidation of erythrocytes; GSH—glutathione; SOD—superoxide dismutase; CAT—catalase; MDA—malondialdehyde; GPX—glutathione peroxidase; TG—triglycerides; ox-LDL—oxidised LDL; ApoB—apolipoprotein B; PON—paraoxonase; Lp(a)—lipoprotein(a); NO—nitric oxide; TAS—total antioxidant status; RGSH—reduced glutathione; NOX—NADPH oxidase; ROS—reactive oxygen species.

**Table 2:** Protective effects of dietary antioxidants on the antioxidative status in patients with psoriasis.

Antioxidant/Design	Duration of Therapy	Characteristic	Target Sites	Antioxidant Capacity	References
Tablet containing 100 μg selenium + 1500 IU vitamin A + 90 mg vitamin C + 30 mg vitamin E; once daily	4 weeks	Seven psoriatic patients, different severities	Plasma, erythrocytes	≈ MDA; ≈ CAT; ≈ SOD	[48]
Epigallocatechin-3-gallate EGCG glycerin solvent (3%, 30 mg/mL, glycerin solvent: 50% glycerin, 50% normal saline)	3 weeks	Imiquimod induced psoriasis-like BALB/c mice	Serum	↑ SOD; ↑ CAT; ↓ MDA	[153]
30 mg β-carotene + 27.5 mg zinc sulfate monohydrate + 0.2 mg monohydrated selenium dioxide + 2 mg manganese + 1 mg copper	8 weeks	33 patients, 20–60 years, chronic plaque psoriasis	Serum	↓ MDA; ↑ TAC	[157]
50 mg/d coenzyme Q10 (ubiquinone acetate) + 50 mg/d vitamin E (natural alpha-tocopherol) + 48 µg/d selenium (aspartate salt) dissolved in soy lecithin	30–35 days	58 patients, severe erythrodermic and arthropathic forms of psoriasis	Granulocytes, affected epidermis	↑ SOD; ↑ CAT	[158]
*Nigella sativa* ointment (20% w/w); *Nigella sativa* capsule (500 mg seeds powder)	12 weeks	60 patients, 3 groups: Group 1 (control)—methotrexate tablets; Group 2—*Nigella sativa* ointment 2 times daily + *Nigella sativa* capsule 3 times daily; Group 3—methotrexate tablets + *Nigella sativa* (ointment + capsule).	Serum	↓ MDA	[160]

↑—Increased concentration or activity in comparison to the control (untreated) group; ↓—decreased or inhibited concentration or activity in comparison to the control (untreated) group; ≈ no differences in comparison to the control (untreated) group; TAC—total antioxidant capacity; MDA—malondialdehyde; SOD—superoxide dismutase; CAT—catalase.
